# Homeodomain-Interacting Protein Kinase (HIPK)-1 Is Required for Splenic B Cell Homeostasis and Optimal T-Independent Type 2 Humoral Response

**DOI:** 10.1371/journal.pone.0035533

**Published:** 2012-04-24

**Authors:** Fiona M. Guerra, Jennifer L. Gommerman, Steven A. Corfe, Christopher J. Paige, Robert Rottapel

**Affiliations:** 1 Department of Immunology, University of Toronto, Toronto, Ontario, Canada; 2 Ontario Cancer Institute, Toronto, Ontario, Canada; 3 Ontario Institute for Cancer Research, Toronto, Ontario, Canada; 4 Department of Medical Biophysics, University of Toronto, Toronto, Ontario, Canada; 5 St. Michael's Hospital, Toronto, Ontario, Canada; 6 University Health Network, Toronto, Ontario, Canada; University Paris Sud, France

## Abstract

The homeodomain-interacting protein kinase (HIPK) family is comprised of four highly related serine/threonine kinases originally identified as co-repressors for various homeodomain-containing transcription factors. The HIPKs have been shown to be involved in growth regulation and apoptosis, with numerous studies highlighting HIPK regulation of the tumor suppressor p53. In this study, we have discovered a B cell homeostatic defect in HIPK1-deficient (*HIPK1^−/−^*) mice. Lymphopoietic populations within the thymus and bone marrow of *HIPK1^−/−^* mice appeared normal based upon FACS analysis; however, the spleen exhibited a reduced number of total B cells with a significant loss of transitional-1 and follicular B cell populations. Interestingly, the marginal zone B cell population was expanded in *HIPK1^−/−^* mice, yielding an increased frequency of these cells. *HIPK1^−/−^* B cells exhibited impaired cell division in response to B cell receptor cross-linking *in vitro* based upon thymidine incorporation or CFSE dilution; however, the addition of CD40L rescued *HIPK1^−/−^* proliferation to wild-type levels. Despite the expanded MZ B cell population in the *HIPK1^−/−^* mice, the T-independent type 2 humoral response was impaired. These data identify HIPK1 as a novel kinase required for optimal B cell function in mice.

## Introduction

The murine splenic B cell population is a heterogeneous population comprised of developing B cells as well as subsets of mature B cells. While the vast majority of splenic B cells are follicular (FO), only 5–10% are marginal zone (MZ) B cells [1], [2]. Broadly speaking, FO B cells respond to thymus-dependent (TD) antigens, however, they recently have been shown to also participate in T cell-independent responses in the bone marrow (BM) [3], [4]. MZ B cells are localized near the marginal sinus, between the white and red pulp, and are thus in a prime location to function as the first line of defense against blood-borne pathogens [2], [5], [6]. MZ B cells produce natural antibodies, and resemble memory cells in that they have an activated phenotype, they self-renew and have an unlimited lifespan. FO B cells, in contrast, have a lifespan of weeks. The mechanisms underlying the fate decisions controlling FO and MZ development remain elusive. MZ B cell development requires Delta-like 1 (DL1) and B cell activating factor (BAFF) signaling, as well as chemotactic and integrin signaling (reviewed [6]). Several studies have also identified a role for B cell receptor (BCR) signal strength in determining the FO versus MZ fate decision [2], [7], [8].

Two hypotheses have emerged to explain what drives commitment to the MZ B cell fate: the “production bottleneck” hypothesis and the “signal strength” hypothesis. The splenic MZ population is preferentially maintained in the absence of B cell influx from the BM [9], [10], [11], and several genetic mouse models have reported enlarged splenic MZ compartments in the context of impaired early B lymphopoiesis [2]. The “production bottleneck” hypothesis conjectures that this phenomenon arises as a compensatory mechanism that favours the development of the effector branch of the B cell system when B lymphopoiesis is impaired [2]. MZ B cells are considered to be the effector branch due to their activated phenotype and their ability to rapidly produce natural IgM. In contrast, the “signal strength” hypothesis argues that the strength of the BCR signal regulates commitment to the FO and MZ B cell fates [2], [7], [8]. Weak BCR signaling preferentially commits developing B cells to the MZ B cell fate, whereas strong BCR signals favour the FO B cell fate [2], [6].

The homeodomain-interacting protein kinase (HIPK) family is comprised of four evolutionarily conserved and highly related nuclear serine/threonine kinases [12], [13]. Structurally, HIPKs possess a homeoprotein-interaction domain, kinase domain, PEST domain, a tyrosine/histidine-rich (YH domain) C-terminus, as well as phosphorylation and sumoylation sites [14]–[16]. HIPKs 1–3 were originally identified as co-repressors for various homeodomain-containing transcription factors [13]. HIPK4 was discovered in the human genome sequence based on its high homology to the other members of the HIPK family [12]. HIPK4 is a truncated version of the kinase, which lacks the homeoprotein-interaction domain making it 616 amino acids, and is primarily cytoplasmic in its localization [17], [18]. The HIPKs interact with a variety of proteins involved in regulating cellular stress responses. During the DNA damage response HIPK2 phosphorylates Ser46 of p53, which facilitates cyclic AMP response element-binding (CREB)-binding protein (CBP)-mediated acetylation of p53 at Lys382, leading to p53-dependent gene expression [19]–[21]. In addition to p53, HIPK2 interacts with several other proteins involved in apoptosis and proliferation, including p63 and p73 [19], [20], [22], Brn3a [23], c-Ski [24], CtBP [25], [26], and components of the Wnt pathway [27]–[35]. Like HIPK2, HIPK1 is also a p53 kinase, however the site of phosphorylation is unknown [12], [36]. In addition to modulation of p53-induced apoptosis, HIPK1 has been implicated in controlling apoptosis signal-regulating kinase (ASK)-1-induced apoptosis [16], [37], [38]. These studies suggest that at least one of the functions of the HIPK family is to regulate growth and apoptosis pathways. Deletion of either HIPK1 or HIPK2 does not affect viability in mice; however, deletion of both HIPK1 and HIPK2 is embryonic lethal with fetal death at embryonic day 9.5–12.5 due to a neural tube closure defect (NTD), demonstrating a functional requirement for HIPK activity in developing tissues [39]. The high expression of HIPK1 in cells of the hematopoietic lineage relative to non-hematopoietic cells suggests HIPK1 may play a significant role in hematopoietic cell development and/or function.

In the present report, we have analyzed the role of HIPK1 in the B cell compartment using HIPK1-deficient (*HIPK1^−/−^*) mice [36]. Our phenotypic analysis of *HIPK1^−/−^* mice has revealed a splenic B cell homeostatic defect resulting in a reduced number of total splenic B cells, but an increase in the MZ B cell population. Despite the expanded MZ B cell population, *HIPK1^−/−^* mice exhibited a significantly impaired T-independent type 2 (TI-2) humoral response. Based on our studies, we have identified a requirement for HIPK1 in splenic B cell homeostasis and activation.

## Materials and Methods

### Ethics Statement

Animals were maintained in pathogen-free conditions at the Ontario Cancer Institute and the Max-Bell Institute under University Health Network Animal Resource Centre guidelines (permit 1292.8).

### Mice

*HIPK1^−/−^* mice were a gift from T.W. Mak (Campbell Family Cancer Research Institute and Ontario Cancer Institute, Toronto, ON) and have been described previously [36]. *HIPK1^−/−^* mice were backcrossed to C57/Black6 one additional generation to generate heterozygous offspring, which were then intercrossed to obtain *HIPK1^+/+^* and *HIPK1^−/−^* mice for further propagation and analysis. Mice were age- and sex-matched for all experiments. *HIPK1^+/+^* and *HIPK1^−/−^* genotypes were determined by polymerase chain reaction (PCR) amplification of tail DNA. Tail clippings were digested overnight at 55°C in a digesting solution containing 10 mM EDTA, 20 mM Tris, 10% SDS and 25 µg/ml Proteinase K (Invitrogen, Burlington, ON). DNA was purified using the DNeasy Purification Kit (Qiagen, Mississauga, ON), and diluted 1/10 for use in PCR reactions. The primers used to identify a *hipk1* fragment of ∼350 base pairs were 5′-ACCTGCTGCAAGATTGGTCT-3′ (sense) and 5′-AGTGTCGTCGAGTGCCTTCT-3′ (antisense). To identify a *neo* fragment of ∼150 base pairs the following primers were used: 5′-GGCGCGAGCCCCTGATGCTC-3′ (sense) and 5′-TTGGGTGGAGAGGCTATTCGGCTATGAC-3′ (antisense). The *hipk1* PCR reaction was as follows: Step 1: 94°C for 5 min. Step 2, repeated 30 cycles: 94°C for 30 s, 61°C for 1 min., 72°C for 1 min. Step 3: 72°C for 5 min. The *neo* PCR reaction was as follows: Step 1: 94°C for 5 min. Step 2, repeated 30 cycles: 95°C for 1 min., 62°C for 1 min., 72°C for 1 min. Step 3: 72°C for 5 min. PCR products were run on a 1.5% agarose gel. Mice age ranged from 8 to 12 weeks.

### Chemicals

Unless otherwise indicated, all chemicals were purchased from Sigma.

### HIPK1 expression in lymphocytes

Primary B cells were purified from the spleens of *HIPK1^+/+^* and *HIPK1^−/−^* mice using the autoMACS system (described below). Total RNA was extracted using an RNeasy Kit (Qiagen, Mississauga, ON) and reverse transcribed to cDNA using random hexamers. Serial dilutions of the cDNA were used for the PCR reactions. The primers used to generate the *hipk1* fragment were described in the genotyping protocol (see above). The primers for *gapdh* amplification were 5′-ACCACAGTCCAT GCCATCAC-3′ (sense) and 5′-TCCACCACCCTGTTGCTGTA-3′ (antisense). The PCR programs were as follows: *hipk1* (see above). *gapdh*: Step 1: 94°C for 5 min. Step 2, repeated for 27 cycles: 94°C for 40 s, 56°C for 45 s, 72°C for 40 s. Step 3: 72°C for 10 min. The PCR products were subjected to electrophoresis on 1% agarose gels.

### Primary lymphocyte isolation and culture

Peritoneal cells were recovered by injecting 5 ml of PBS into the peritoneum of an euthanized mouse and gently massaging the peritoneal cavity before recovering the solution and cells. T cell progenitors were recovered from the thymus of euthanized mice. Single cell suspensions were prepared for cell counting and for FACS analysis using a 70 µM mesh filter (VWR, Mississauga, ON). B lineage progenitors were recovered from the BM of tibia and femurs from euthanized mice using a 26 gauge needle to flush out the BM. Single-cell suspensions were prepared from BM or spleens, and the red blood cells (RBCs) were lysed in 1 ml/spleen ACK solution (155 mM NH_4_Cl, 10 mM KHCO_3_, 0.1 mM EDTA, brought to pH 7.0 using 1 N HCl). Cells were maintained at 37°C in a humidified atmosphere containing 5% CO_2_ during stimulations.

Magnetic cell sorting on the autoMACS Separator (Miltenyi Biotec, Auburn, CA) was used to enrich the B cell population using the B cell Purification Kit according to the manufacturer's instructions (Miltenyi Biotec). Briefly, ACK-treated splenocytes were incubated with a cocktail of biotin-conjugated antibodies against CD43, CD4 and Ter119 for 15 min. at 4°C. Anti-biotin microbeads were then added and incubated for an additional 15 min. at 4°C. Cells were then washed and resuspended with the provided buffers. The microbeads bound to the magnetic column and prevented any splenocytes expressing CD43, CD4 or Ter119 from passing though. The depletion of these cells yielded 98% CD19^+^ cells in the subsequent flow-through. Purified B cells were cultured in RPMI medium (Invitrogen) containing 10% fetal bovine serum (FBS), 50 µM beta-mercaptoethanol, 50Units/L penicillin, 50 mg/L streptomycin and 10 mM HEPES. Cells were maintained at 37°C in a humidified atmosphere containing 5% CO_2_ during stimulations.

### Flow cytometry

For flow cytometry the following biotin-, FITC-, phycoerythrin (PE)-, (PerCP)- and allophycocyanin (APC)-labeled antibodies were purchased from BD Biosciences (San Diego, CA): anti-B220 (RA3-6B2), anti-CD43 (S7), anti-BP.1 (6C3), anti-HSA (M1/69), anti-CD4 (GK1.5), anti-CD8 (53-6.72), anti-CD25 (7D4), anti-CD44 (1M7), anti-CD3 (145-2C11), anti-IgD (SBA.1), anti-CD21 (7G6), anti-CD23 (B3B4), CD1d (1B1) and AnnexinV. Anti-IgM (II/41; R6-60.2) was purchased from eBioscience. Cells were washed in FACS Solution (PBS, 1% FCS and 0.1% NaN_3_), and incubated at 5×10^7^ cells/ml with FACS antibodies for 25 min. at 4°C. After staining, cells were washed twice in FACS Solution, and resuspended in approximately 400 µl of FACS Solution. In some staining, propidium iodide (PI)(Sigma) or 7-amino-actinomycin D (7AAD)(Sigma) were used to identify dead cells. All samples were analyzed on a FACSCalibur instrument (BD, Mississauga, ON) and the data was then analyzed using FloJo software (Tree Star, Ashland, OR).

To detect caspase-3 positive T1 B cells, total splenocytes were isolated from *HIPK1^+/+^* and *HIPK1^−/−^* mice and immediately incubated with FITC-DEVD-FMK (EMDBiosciences, Mississauga, ON) according to the manufacturer's instructions. The splenocytes were then stained with B220, CD23, and CD21 so that T1 B cells could be identified. 90 000 total events were acquired.

### Antigen receptor activation of primary B cells

Purified B cells were plated at 1×10^6^ cells/ml, and stimulated with F(ab′)_2_ anti-mouse IgM (10 µg/ml; Jackson Immunoresearch, West Grove, PA) with or without CD40L (10 µg/ml; 1C10, Southern Biotech, Birmingham, AL) for the indicated amounts of time.

### Proliferation Assays

For thymidine incorporation assays, cells were pulsed with 1 µCi [^3^H] thymidine 12 hrs before the desired time point. The cells were harvested onto 96-well plates containing fiber filters and the incorporated thymidine was measured using the TopCount liquid scintillation counter (Canberra/Packard, Mississauga, ON). For the cell division assay, Cell Tracker Green 5-chloromethylfluorescein diacetate (CFSE)(Molecular Probes, Burlington, ON) was used according to the manufacturer's recommendations. Briefly, cells were stained in 5 µM CFSE in PBS at 37°C for 5 min. with periodic, gentle swirling. Cells were then resuspended in 4 ml FBS and pelleted at 400× *g* at 4°C. Cells were then washed twice with cold PBS before analysis. FloJo Proliferation Platform (Treestar) was used to analyze the results of the cell division assay.

### Immunoprecipitation and Western Blotting

Cell pellets were lysed in a kinase lysis buffer (20 mM Tris-HCl pH7.5, 150 mM NaCl, 1 mM EDTA, 0.5% sodium deoxycholate, 1% NP-40), containing fresh 1 mM sodium orthovanadate, 50 mM NaF, 1 mM phenylmethylsulfonyl fluoride and 1× ETDA-free protease inhibitor cocktail (Roche, Laval, PQ) for 10 min. on ice. The lysates were then clarified by centrifugation at 16, 000× *g* at 4°C. For immunoprecipitation, lysates were first pre-cleared with Protein G beads (Invitrogen) for 45 min. on a nutator. The pre-cleared lysates were then incubated with the capturing antibody for 2 hrs at 4°C on a nutator. Beads that had been blocked with PBS containing 1% BSA were then added to the lysates for one hour. The beads were washed three times with kinase lysis buffer, then bound proteins were eluted using 2× sample buffer and boiling at 95°C for five min. The lysates and immunoprecipitates were resolved on 8–10% SDS-PAGE and transferred to Immobilon membranes (Milipore, Jaffrey, NH). Anti-Actin antibody was purchased from Sigma. Antibodies for phospho-Akt (Ser473), Akt, phospho-Erk1/Erk2, Erk1/Erk2, PLCγ, phospho-Btk, Btk, and Syk were purchased from Cell Signaling Technology (Danvers, MA). 4G10 antibodies were purified from hybridoma supernatant and used to detect phospho-tyrosine residues. Immunoreactive proteins were detected using the ECL and ECLplus detection systems (GE Healthcare, UK).

### Immunohistochemistry

Spleens were removed and snap-frozen in OCT compound (Thermo Shandon, Pittsburgh, PA). Sections were generated using a Leica 3050S cryostat (Leica, Wetzlar, Germany), and frozen sections were fixed in ice-cold acetone for 10 min. Spleen sections were first incubated with blocking solution (10% rabbit serum, 10% mouse serum, “Fc block” 2.4G2 antibodies in Tris-buffered saline-0.05% Tween 20 (Sigma)) and then stained with biotinylated anti-IgM (Vector Laboratories Inc, Burlington, ON) and fluorescein isothiocyanate (FITC)-anti-IgD (eBioscience, San Diego, CA) or FITC-anti-MAdCAM1 (MECA-367)(eBioscience). Sections were then stained with the secondary antibodies streptavidin-conjugated horseradish peroxidase (HRP) (Prozyme, San Leandro, CA) and anti-FITC-conjugated alkaline phosphatase (Roche Diagnostics Canada). The sections were developed using the Vector HRP development kit, followed by the Vector alkaline phosphatase substrate kit III, both as per the manufacturer's instructions (Vector Laboratories Inc). Sections were mounted with Crystal/Mount (Biomeda Corp., Foster City, CA) and visualized on a Leica upright DMRA2 microscope.

### Immunization and ELISA

For the trinitrophenylated-Ficoll (TNP-Ficoll; Biosearch Technologies, Novato, CA) immunization, 25 µg of TNP-Ficoll dissolved in phosphate buffered saline (PBS) were administered intraperitoneally (i.p.) to age-matched, male *HIPK1^+/+^* and *HIPK1^−/−^* mice. Blood was collected at the indicated time points by lateral tail-vein bleeds, and serum was isolated using Gel Activator serum separation tubes (Sarstedt, Montreal, PQ). To measure the TI-2 humoral response, serum from TNP-Ficoll-immunized mice was incubated on ELISA plates coated with TNP-BSA. TNP-specific IgM and IgG3 antibodies were detected using secondary anti-IgM-HRP and anti-IgG3-HRP detection antibodies (Southern Biotech). To detect basal serum Ig, plates were coated with a capturing anti-mouse Ig overnight. Serial dilutions of serum were then incubated overnight, and detected the following day using HRP-conjugated anti-IgG1, -IgG2b, -IgG2c, -IgG3, -IgA or –IgM (Southern Biotech).

### Elispot

Age- and sex-matched mice were immunized i.p. with 25 µg TNP-Ficoll. Seven days later, the spleens were harvested and single-cell suspensions were prepared. After RBC lysis, the cells were counted using a hemocytometer and, starting with an equal concentration of cells in the *HIPK1^+/+^* and *HIPK1^−/−^* samples, serial dilutions were plated on TNP15-BSA-coated PVDF-bottomed plates. The plates had been coated with TNP15-BSA overnight at 4°C and then blocked with 5% BSA for three hrs at 37°C prior to plating the splenocytes. The cells were incubated in the plates for 3 hrs at 37°C with no movement. The plates were then washed to remove all cells and cell debris and then incubated with isotype-specific HRP-conjugated antibodies (see above) for one hr at room temperature. The plates were then washed and AEC Chromogen (BD Biosciences) was added as the HRP substrate in order to generate an insoluble precipitate. Finally, the plates were air-dried and maintained in the dark until they were imaged and analyzed using a Cellular Technology Ltd (CTL) Immunospot plate reader (CTL, Shaker Heights, OH).

### Statistics

To calculate p values, data were analyzed by the unpaired student t test unless otherwise indicated. *p≤0.05, **p≤0.01, ***p≤0.001.

## Results

### HIPK1 is expressed in cells from the hematopoietic lineage

**Figure 1:**
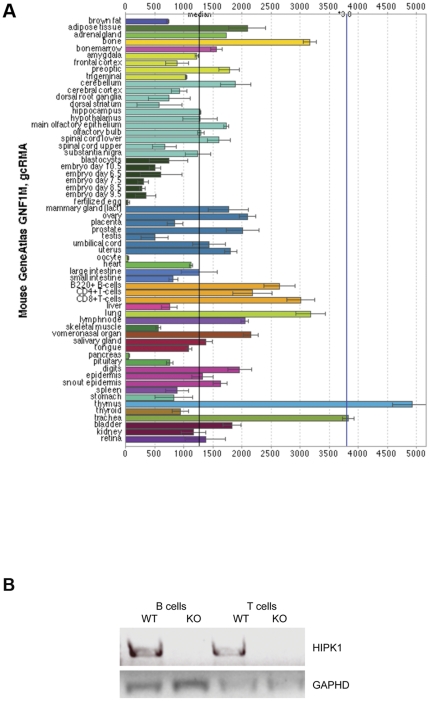
HIPK1 is expressed in hematopoietic cells. A, Analysis of the tissue-specific gene expression profiles of *hipk1* and *hipk2* using the BioGPS-Gene Portal Hub web application (Genomics Institute of the Novartis Research Foundation (GNF). For this study, B and T lymphocytes were obtained from spleen. B, Primary B and T lymphocytes were isolated from the spleens of *HIPK1^+/+^* (WT) and *HIPK1^−/−^* (KO) mice. mRNA was isolated and reverse transcribed to cDNA. PCR amplification was used to determine if *hipk1* was expressed. *gapdh* was used as a control.

Analysis of the tissue-specific gene expression profiles of *hipk1* and *hipk2* using the BioGPS-Gene Portal Hub web application (Genomics Institute of the Novartis Research Foundation (GNF) revealed that *hipk1* is highly expressed in hematopoietic cells (Figure 1A). We verified the expression of *hipk1* in purified primary B cells and T cells by PCR amplification of cDNA (Figure 1B). *hipk1* expression was detected in wild-type primary B and T cells, but was absent in *HIPK1^−/−^* samples.

### HIPK1 is not required for T cell development in the thymus or B cell development in the BM

**Figure 2:**
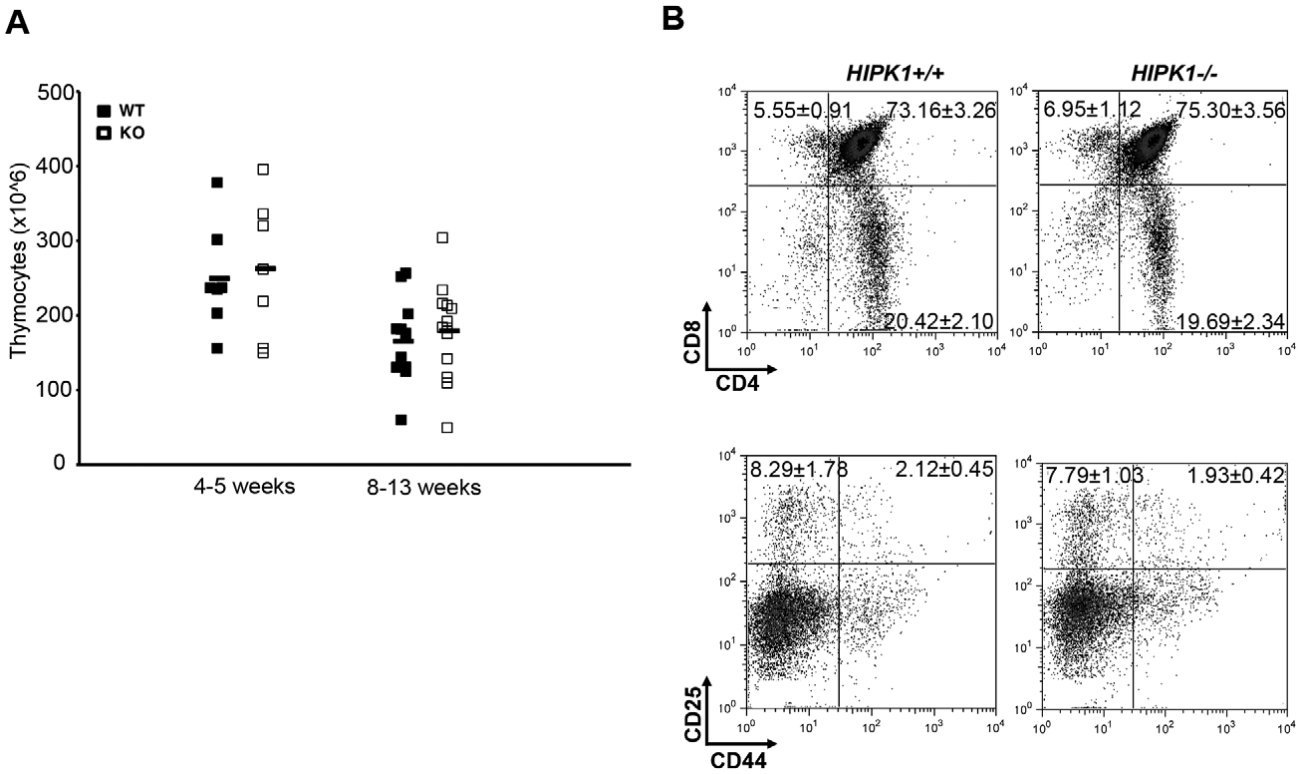
*HIPK1**^−/−^*** mice exhibit normal T cell development in thymi. A, Thymic cellularity was similar between *HIPK1^+/+^* and *HIPK1^−/−^* mice at 4–5 weeks and 8–13 weeks. For 4–5 week old mice, n = 7; for 8–13 week old mice, n = 13. B, CD4 and CD8 expression was used to identify the frequency of double-positive (DP) and single positive populations, and did not reveal any abnormalities. FACS analysis of thymocyte subsets using CD44 and CD25 expression indicated that the double-negative DN I–IV populations were intact in *HIPK1^−/−^* mice. Representative FACS plots are shown (n = 9).

**Figure 3:**
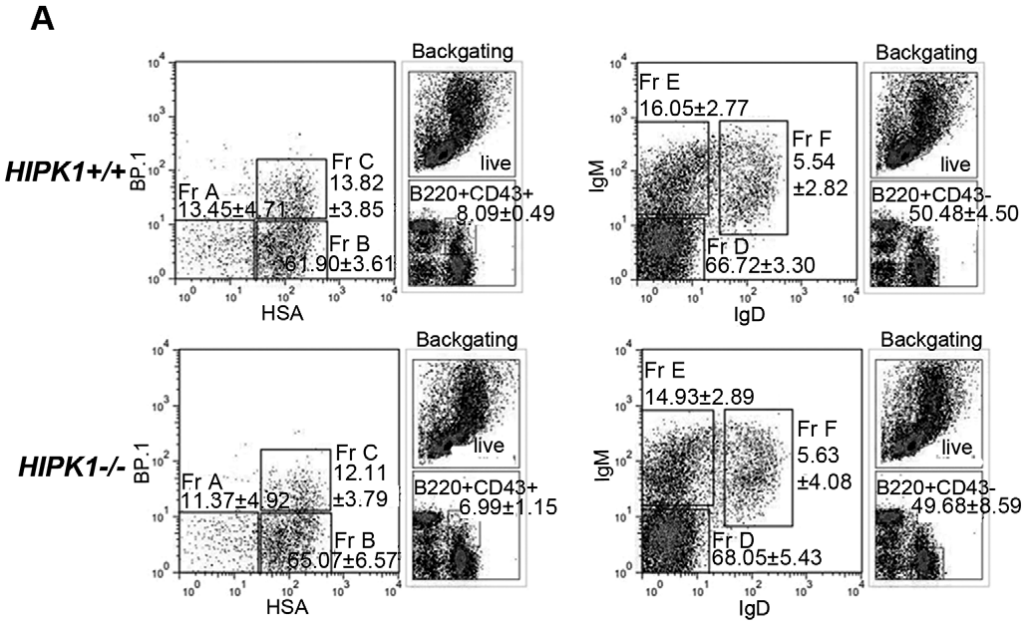
*HIPK1**^−/−^*** mice exhibit normal B cell development in the bone marrow. A, FACS analysis of bone marrow. Bone marrow was isolated, ACK-treated and stained with B220, IgM, BP.1, CD43, HSA and IgD in order to determine the frequencies of developing B cell subsets; representative FACS plots are shown (n = 8).

Based upon the high expression of HIPK1 in lymphocytes (Figure 1A), lymphocyte subsets from primary lymphoid organs of *HIPK1^−/−^* mice were analyzed for developmental abnormalities. Thymic cellularity was normal in *HIPK1^−/−^* mice (Figure 2A). FACS analysis of thymocyte subsets using CD4, CD8, CD44, and CD25 expression indicated that the double-negative I–IV, double-positive and single-positive populations were intact in the *HIPK1^−/−^* mice, (Figure 2 A, B). Although there was a subtle increase in the CD8 single-positive population in the *HIPK1^−/−^* mice, this difference was not statistically significant. To analyze the frequency of B cell progenitor populations in the BM, gated B220^+^ cells were separated into either CD43^+^ or CD43^−^ populations (Figure 3A). B220^+^CD43^+^ cells were then analyzed for the expression of HSA and BP.1 to determine relative frequencies of Hardy fractions (Fr.) A (HSA^−^BP.1^−^), Fr. B (HSA^+^BP.1^−^) or Fr. C (HSA^+^BP.1^+^). B220^+^CD43^−^ cells were analyzed for the expression of IgM and IgD to determine relative frequencies of Hardy Fr. D (IgM^−^IgD^−^), Fr. E (IgM^+^IgD^−^), or Fr. F (IgM^+^IgD^+^). These analyses demonstrated that there were no developmental abnormalities within the BM B cell lineage as measured by the steady state frequencies of B cell subsets in the *HIPK1^−/−^* derived BM.

### HIPK1 is required for splenic B cell homeostasis

**Figure 4:**
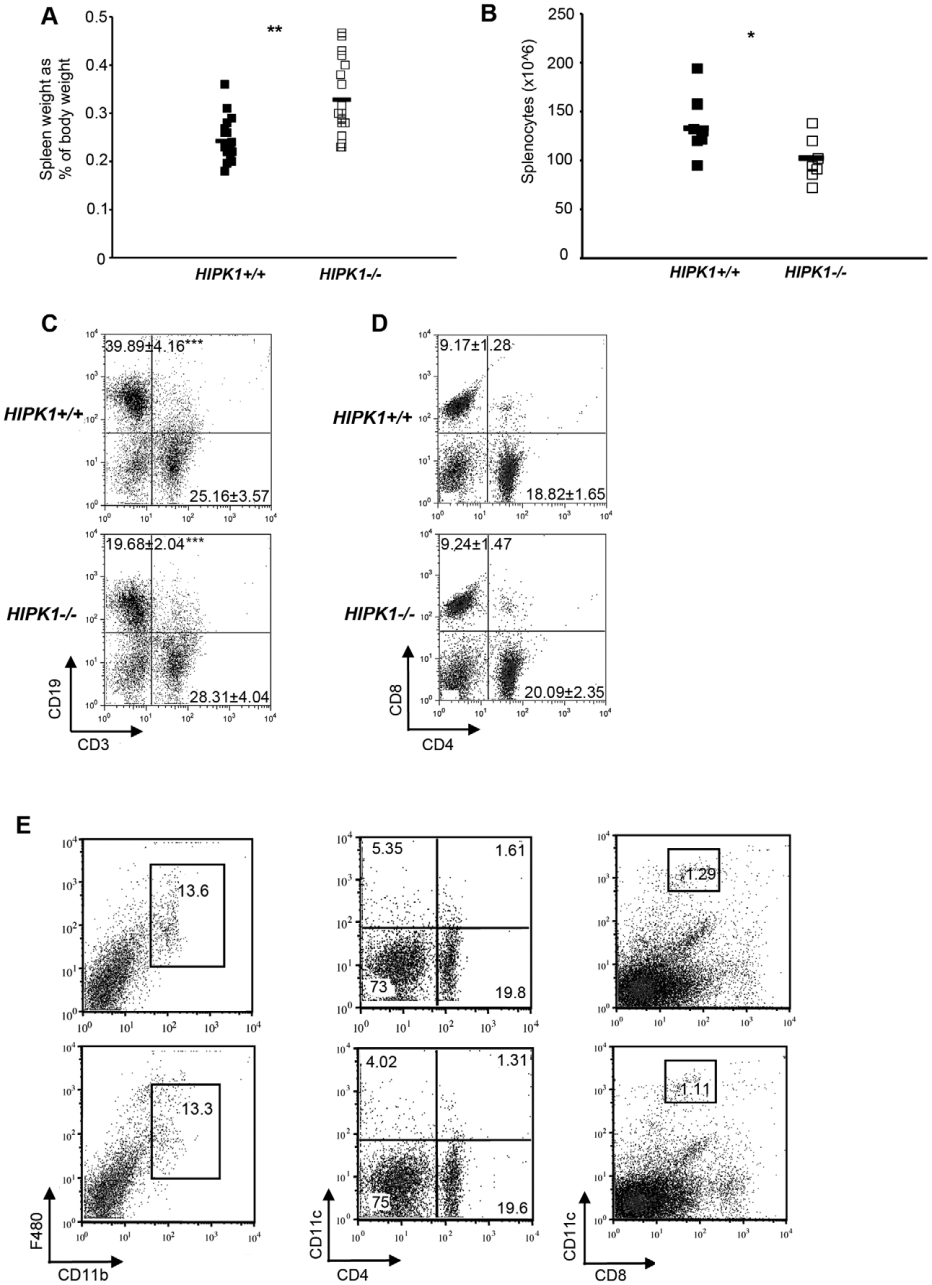
Analysis of spleens from *HIPK1**^−/−^*** mice. A, Mice were weighed after being sacrificed. Spleens were then excised and weighed, and expressed as a percentage of the total mouse weight (n = 16). B, The number of splenocytes obtained after ACK-treatment was counted using a hemacytometer (n = 7). C–D, FACS was used to identify splenic populations. Representative FACS plots are shown; stains were performed in at least three independent experiments with similar results unless indicated otherwise. C, CD3 was used to identify T cells, and CD19 to identify B cells (n = 7). D, CD4 and CD8 were used to identify splenic T cell subpopulations (n = 6). E, CD11b and F480 were used to identify macrophages. CD11c, CD4 and CD8 were used to identify dendritic cells (n = 3). *p≤0.05, **p≤0.01, ***p≤0.001.

Mild splenomegaly was observed in the absence of HIPK1. When spleen weight was expressed as a percentage of total body weight, wild-type mice yielded an average value of 0.242%±0.011 compared to *HIPK1^−/−^* mice which yielded an average value of 0.328%±0.019 (Figure 4A). RBC lysis resulted in a reduction in the overall cellularity of *HIPK1^−/−^* spleens, 102×10^6^±10.74 cells/spleen compared to 133×10^6^±8.38 cells/spleen in wild-type controls (Figure 4B). The present study demonstrates that in the absence of HIPK1, adult mice exhibit evidence of extramedullary erythropoiesis. Hattangadi *et al*. have recently demonstrated that HIPK2 plays an important role in terminal erythroid differentiation [40], supporting a role for the HIPK family in erythroid differentiation.

To investigate the cause of the reduced splenic cellularity, the splenic lymphocyte populations were analyzed by flow cytometry. The percentage of CD19^+^ splenocytes was reduced in *HIPK1^−/−^* mice, resulting in 19.68±2.04% CD19+ cells compared to 39.89±4.16% in the *HIPK1^+/+^*mice (Figure 4C). The percentage of CD3+ splenocytes was subtly increased in the *HIPK1^−/−^* mice, but no statistically significant differences were observed compared to wild-type controls (Figure 4C). The percentage of splenic CD4^+^ and CD8^+^ T cells was also similar between *HIPK1^−/−^* mice and wild-type controls (Figure 4D). The percentage of macrophages, based on F480 and CD11b staining, was similar between *HIPK1^−/−^* and wild-type mice (Figure 4E). Using FSC, SSC, CD11c, CD4 and CD8 as dendritic cell markers also revealed no abnormalities (Figure 4E). These observations suggest that the reduced splenic cellularity in the *HIPK1^−/−^* was largely due to decreased splenic B cells.

**Figure 5:**
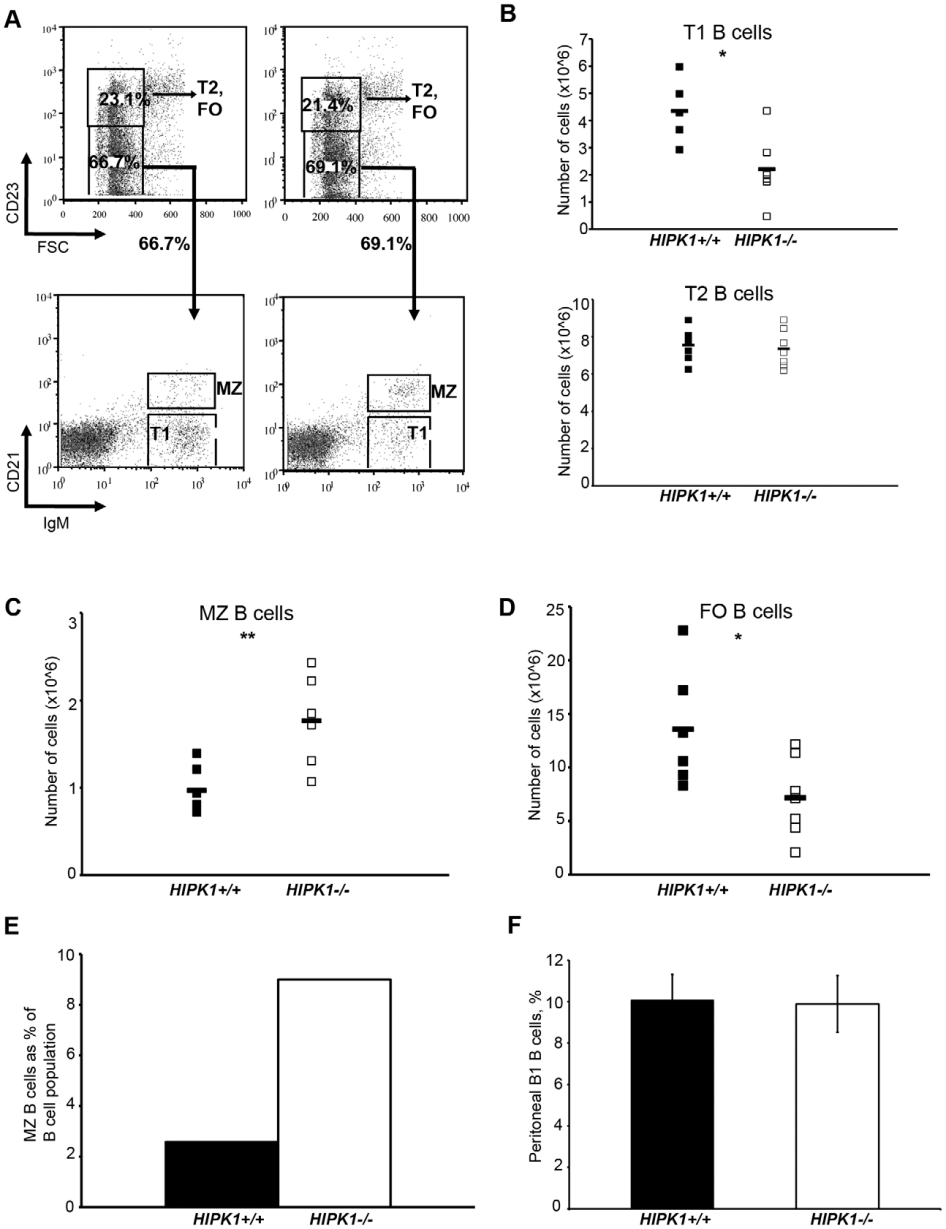
Splenic B cell homeostasis is disrupted in *HIPK1**^−/−^*** mice. A, Splenic B cells were stained using CD23, CD21, and IgM to distinguish between B cell subsets. Representative FACS plots are shown for experiments that were conducted at least three times with similar results. B–D, Frequencies obtained by FACS were converted to absolute numbers and averaged. B, The absolute number of T1 B cells was reduced in *HIPK1^−/−^* mice, whereas the absolute number of T2 B cells was unaffected (n = 6). C, The absolute number of MZ B cells was elevated in *HIPK1^−/−^* mice (n = 6). D, The absolute number of FO B cells was reduced in *HIPK1^−/−^* mice (n = 7). E, The MZ B cell population expressed as a percentage of the total B cell population. F, Cells obtained from peritoneal lavage were stained with CD5 and IgM to identify the B1 B cell population by FACS (n = 6). *p≤0.05, **p≤0.01.

**Figure 6:**
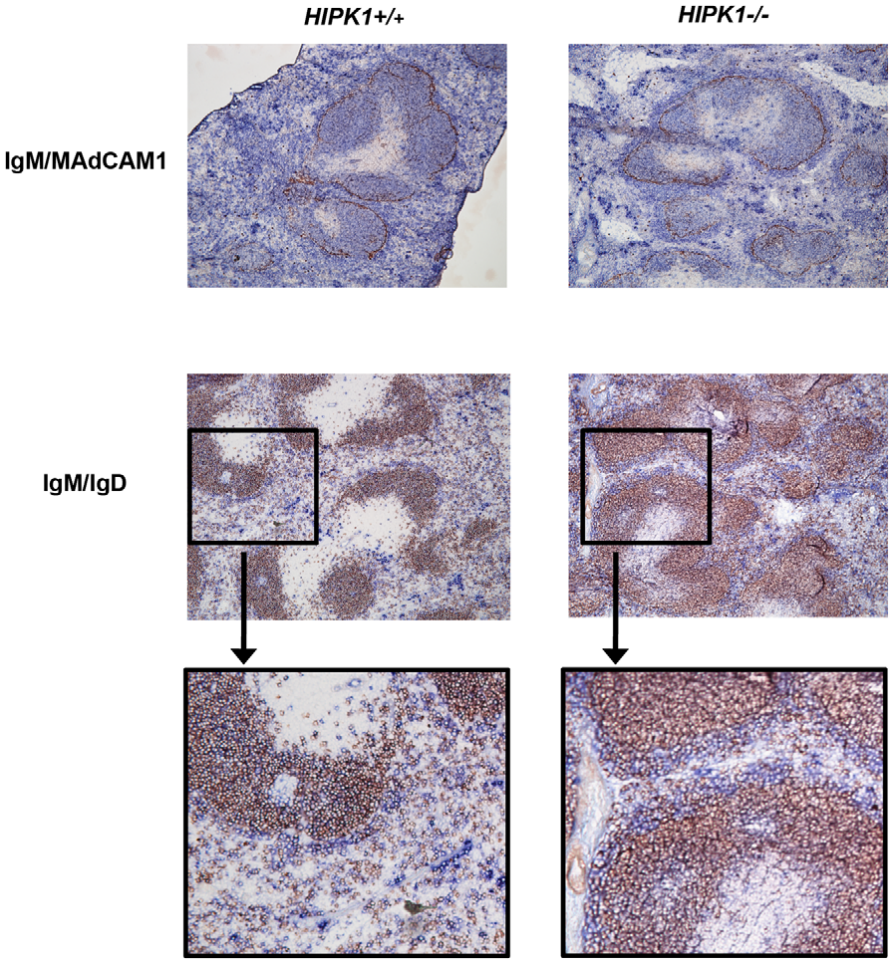
Enlarged MZ compartment in *HIPK1**^−/−^*** mice. Splenic sections were stained with IgM (blue) and IgD (red) or MAdCAM1 (red) to identify the MZ B cell population and the marginal sinus. Representative sections are shown, with similar results observed in three mice.

Splenic B cells can be subdivided into transitional (T)-1 and T–2, FO, and MZ B cells by differential surface expression of CD23, CD21, IgM and IgD. FACS analysis of *HIPK1^−/−^* splenocytes using FSC, CD23, CD21 and IgM surface markers revealed a reduced percentage of T1 B cells and a 2.2-fold decrease in the absolute number of T1 B cells compared to wild-type mice (Figure 5A, B). This decrease was not attributable to decreased viability of the T1 population (Figure S1). The T2 B cell population, however, was unaffected by the absence of *HIPK1* (Figure 5B). In contrast, *HIPK1^−/−^* mice exhibited an increase in the MZ B cell population, which translated into a 1.9-fold increase in their absolute numbers compared to wild type mice (Figure 5C). The mature fraction of FO B cells was reduced by 1.6-fold in the *HIPK1^−/−^* mice (Figure 5D). Due to the reduced absolute number of splenic B cells in the *HIPK1^−/−^* mice, the 1.9-fold increase in the absolute number of MZ B cells resulted in a 3.5-fold higher frequency of MZ B cells (Figure 5E). Immunohistochemistry of splenic sections stained with IgM and IgD revealed an expansion of the MZ in the *HIPK1^−/−^* mice compared to wild-type mice, consistent with our observation that MZ B cell numbers are increased in *HIPK1^−/−^* mice (Figure 6). Analysis of peritoneal B1 B cells revealed no difference in the percentage of B1 B cells between *HIPK1^−/−^* and wild-type mice (Figure 5F). Thus, HIPK1 is specifically required for normal B cell homeostasis in the spleen.

**Figure 7:**
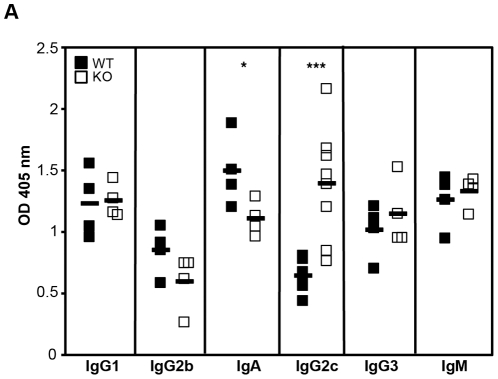
Basal serum Ig levels. A, Basal serum Ig levels from 12 week-old *HIPK1^+/+^* and *HIPK1^−/−^* mice were measured by ELISA. n = 4, except for IgG2c, where n = 8. *p≤0.05, **p≤0.01.

Based upon the disruption of the steady-state levels of the splenic B cell populations, we investigated the basal serum immunoglobulin (Ig) levels. Unstimulated adult *HIPK1^−/−^* mice had similar basal levels of IgM, IgG1 and IgG3 compared to wild-type controls (Figure 7). In contrast, IgA and IgG2b levels were lower in *HIPK1^−/−^* mice compared to wild-type controls (Figure 7A). It has been reported that MZ B cells switch to IgG2b and IgA, with a particular propensity towards IgA compared to FO B cells [41]. Thus, the reduced IgA and IgG2b levels in *HIPK1^−/−^* mice may be a reflection of impaired MZ B cell function, despite the expansion of this population. Interestingly, the elevated IgG2c in the serum of the HIPK1^−/−^ mice was not accompanied by a decrease in the IgG1 levels and increase in IgG3 levels, which is seen in response to IFN-γ. Thus, the elevated IgG2c is unlikely to be the result of cytokine skewing.

### HIPK1 is required for optimal B cell proliferation in response to BCR stimulation

**Figure 8:**
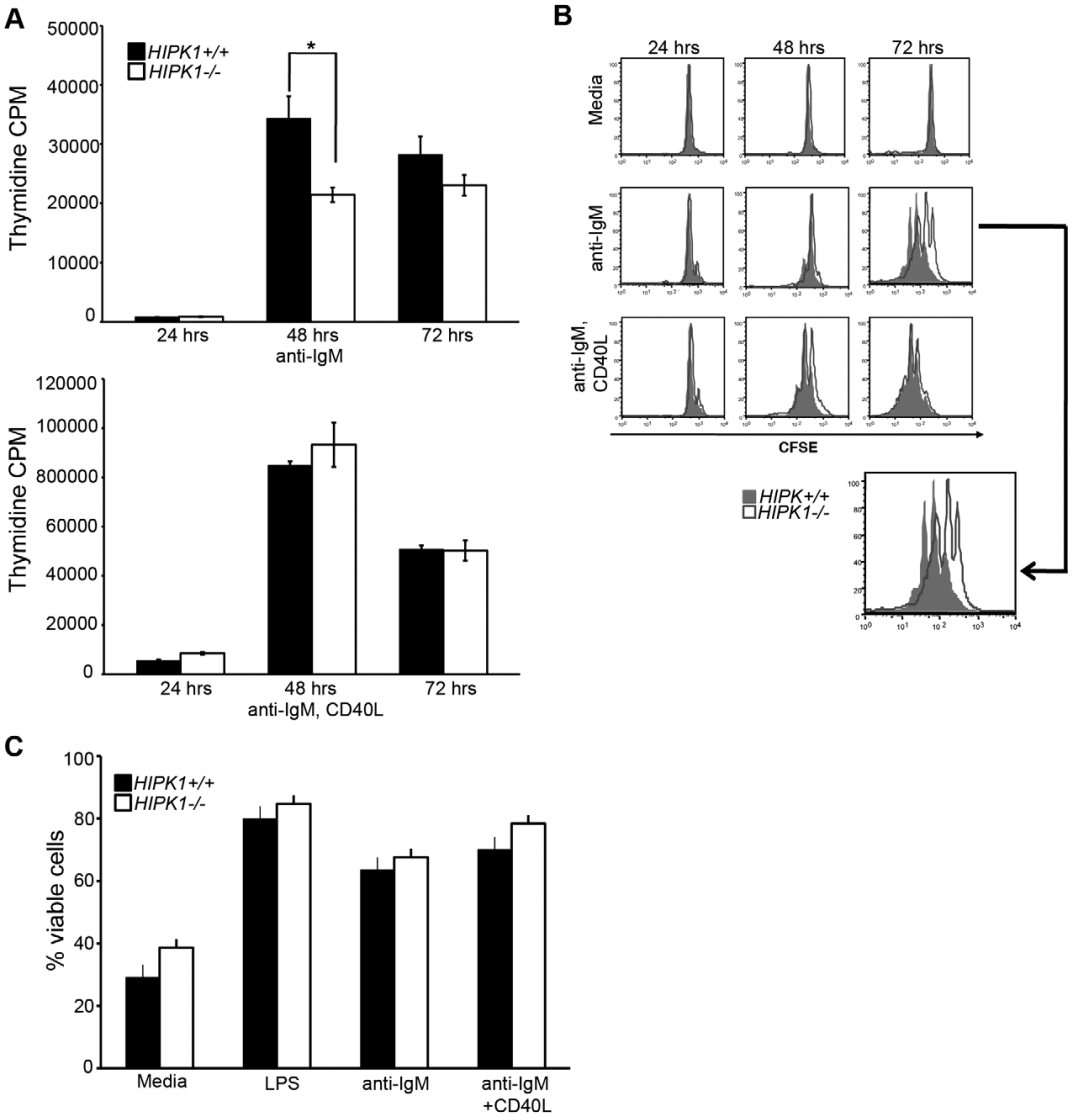
HIPK1 is required for BCR-induced proliferation. A, Proliferation of *HIPK1^+/+^* and *HIPK1^−/−^* splenic B cells in response to anti-IgM ± CD40L (10 µg/ml each) was assessed by [3H]-thymidine incorporation. Cultures were pulsed with 1 µCi tritiated thymidine 12 hrs before the indicated time points. Representative results of three independent experiments are shown. B, Cell division of *HIPK1^+/+^* and *HIPK1^−/−^* splenic B cells was determined by CFSE dilution assay. Cells were stimulated with anti-IgM ± CD40L (10 ug/ml) or media alone, and analyzed at 24, 48, and 72 hrs post-stimulation. The solid peaks are wild type and the empty peaks are *HIPK1^−/−^*. FACS plots are representative of three independent experiments. C, Viability was measured by FACS by gating on the AnnexinV and PI double-negative populations at 48 hrs after stimulation (n = 4). *p≤0.05.

The decreased number of splenic B cells in *HIPK1^−/−^* mice prompted us to investigate the responsiveness of *HIPK1^−/−^* B cells via BCR stimulation. Resting B cells were purified by negative selection, yielding 98% B cell purity by B220 or CD19 staining (not shown). The cells were then stimulated with anti-IgM ± CD40L, or media alone for different intervals and then pulsed with [^3^H] thymidine. *HIPK1^−/−^* B cells exhibited impaired proliferation in response to BCR stimulation (62% of the wild-type response), whereas [^3^H] thymidine incorporation was similar to the wild-type response when CD40L was added (Figure 8A). To determine whether the observed difference in [^3^H] thymidine incorporation was due to impaired cell division, cell division rates were quantitatively measured using the CFSE dilution assay. Wild-type and *HIPK1^−/−^* B cells were loaded with CFSE, stimulated with anti-IgM ± CD40L, and the fluorescence intensity of the dye was measured over time. The viable *HIPK1^−/−^* B cell population exhibited a distinct cell division lag at 72 hours post-stimulation compared to wild-type B cells (Figure 8B). Only 21% of viable *HIPK1^−/−^* B cells had undergone two or more divisions, whereas 48% of wild-type control B cells had undergone two or more divisions (Table 1). The reduced rate of cell division in the *HIPK1^−/−^* response to BCR cross-linking was rescued by CD40 co-stimulation. The viability of *HIPK1^−/−^* B cells following BCR cross-linking was determined by AnnexinV and & 7-AAD staining, and revealed no differences compared to wild-type controls (Figure 8C).

**Table 1:** Impaired proliferation in response to BCR cross-linking.

# of divisions	>2	2	1	0
HIPK1^+/+^	20	28	18	9
HIPK1^−/−^	3	18	30	22

FloJo Proliferation Platform (Tree Star) was used to analyze the percentage of cells having undergone a given number of divisions at 72 hrs in response to anti-IgM stimulation.

### HIPK1 is required for optimal humoral immunity to TI-2 antigen

**Figure 9:**
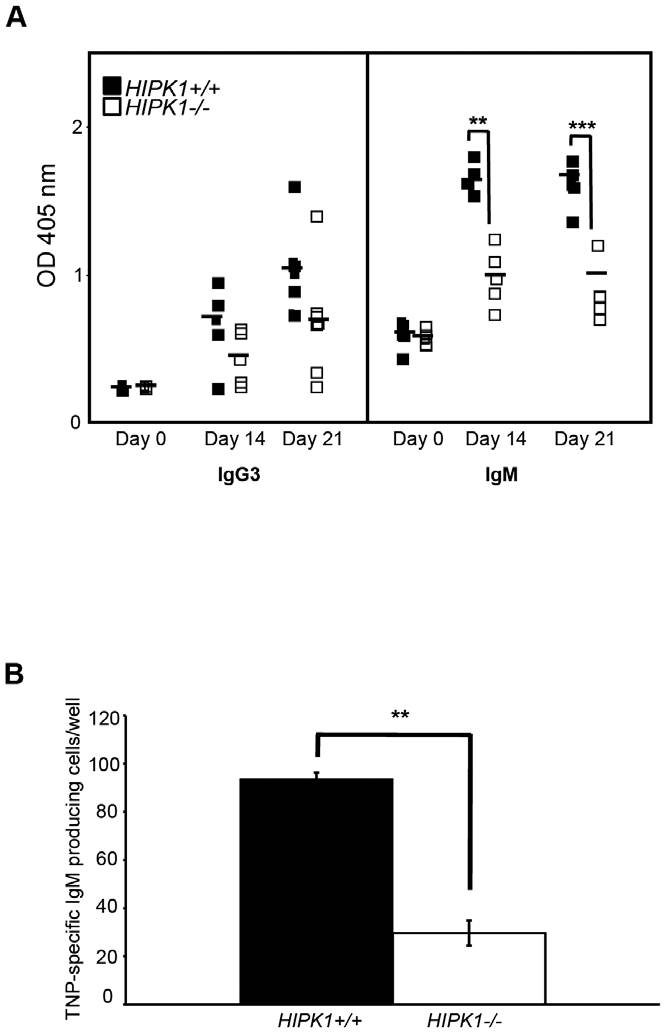
*HIPK1^**−/−**^* mice exhibit an impaired TI-2 humoural response. A, The TI-2 response was evaluated by measuring TNP-specific IgM and IgG3 serum levels after i.p. immunization with 25 µg TNP-Ficoll. Mice were bled at the indicated time points and serum antibody isotypes were measured by ELISA, (n = 5). B, Elispot analysis of TNP-specific-IgM-producing cells in response to TNP-Ficoll seven days post i.p. immunization (n = 4). *p≤0.05, **p≤0.01, ***p≤0.001.

Based upon the disruption of splenic B cell homeostasis in *HIPK1^−/−^* mice, and their impaired response to antigen receptor stimulation *in vitro*, the *in vivo* humoral immune response was analyzed. Wild-type and *HIPK1^−/−^* mice were immunized with the TI-2 antigen TNP-Ficoll, which elicits a largely MZ B cell-dependent response. Following intraperitoneal injection of TNP-Ficoll, serum TNP-specific antibodies were measured by ELISA at days 14 and 21. *HIPK1^−/−^* mice produced dramatically less TNP-specific IgM and IgG3 antibodies compared to wild-type controls, indicating that HIPK1 is required for an optimal humoural response to TI-2 antigen (Figure 9A). In order to enumerate the frequency of antibody producing B cells in *HIPK1^−/−^* spleens following TI-2 antigen immunization, an ELISPOT assay was conducted. Spleens derived from *HIPK1^−/−^* mice had fewer antibody producing cells seven days after immunization compared to spleens from wild-type controls (29.69±5.26 versus 93.69±2.69)(Figure 9B). Thus, fewer *HIPK1^−/−^* B cells were activated and recruited into the response compared to wild-type controls. This, despite *HIPK1^−/−^* mice having more MZ B cells, suggests that *HIPK1^−/−^* MZ B cells poorly respond to antigen receptor stimulation.

### Signaling in the absence of HIPK1

**Figure 10:**
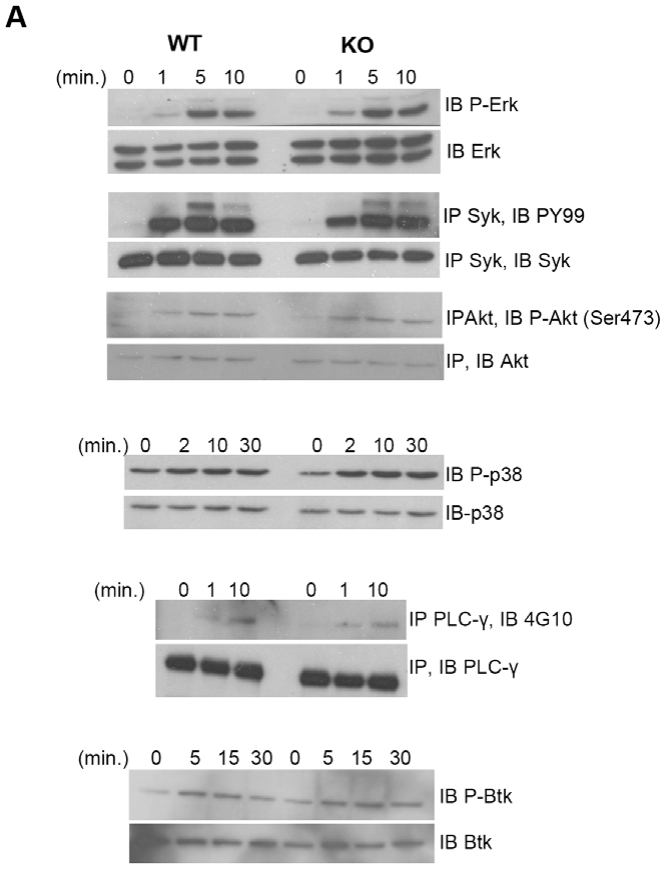
BCR signaling in the absence of HIPK1. A, Primary B cells were stimulated with anti-IgM (10 µg/ml) for the indicated amounts of time and then lysed. Lysates were subjected to SDS-PAGE and were probed with phospho-specific antibodies as indicated. Blots are representative of minimally three independent experiments.

Activation through the BCR results in complex downstream signaling events. To investigate the biochemical defect causing the impaired BCR proliferative response, signaling events following BCR ligation were analyzed in wild-type and *HIPK1^−/−^* B cells. Phosphorylation status was used as an indicator of kinase activation. Anti-IgM-induced phosphorylation of Syk, Btk, Erk, Akt and p38 were unaffected by the absence of HIPK1 (Figure 10A). PLC-γ2 is responsible for the hydrolysis of phosphatidylinositol-4,5-bisphosphate (PIP_2_), yielding inositol-1,4,5-trisphosphate (IP_3_) and diacylglycerol (DAG), which facilitate calcium release and protein kinase C (PKC) activation [42]–[44]. The phosphorylation of PLC-γ2 was also unaffected by the absence of HIPK1 (Figure 10A).

## Discussion

The present study demonstrates that the serine/threonine kinase HIPK1 is required for splenic B cell homeostasis, as well as an optimal TI-2 humoral response. To date, the study of the HIPKs has focused on their role in embryonic development and in regulating cell survival and proliferation. This study is the first to report a role for a member of the HIPK family in lymphocyte function, in particular splenic B cell homeostasis and activation. Our study of *HIPK1^−/−^* mice has revealed a unique role for HIPK1 in splenic B cell homeostasis, a role that cannot be fully compensated for by the other HIPK family members. Other cellular constituents of the spleen including dendritic cells, macrophages, and T cells were not affected by the absence of HIPK1. We did, however, observe evidence of increased extramedullary erythropoiesis in *HIPK1^−/−^* mice. This finding together with the recent report that HIPK2 plays an important role in terminal erythroid differentiation suggest that both HIPK1 and HIPK2 may be required for normal erythropoiesis [40].

The “signal strength” hypothesis argues that the strength of the BCR signal can regulate commitment to the FO versus MZ B cell fate [2], [7], [8]. Strong BCR signaling preferentially commits developing B cells to the FO B cell fate, whereas weak BCR signaling favours the MZ B cell fate [2], [6]. For example, mice deficient in CD22, a negative regulator of BCR signaling, have a reduced number of MZ B cells [45], whereas Aiolos deficient mice have enhanced BCR signaling, and a concomitant reduction in the number of splenic MZ B cells with increased numbers of FO B cells [46], [47]. The Aiolos phenotype can be suppressed by intercrossing the Aiolos-deficient mice with *Xid* mice, which lack a fully functional BTK tyrosine kinase. Attenuation of the BCR signal in these mice restored the number of MZ B cells. These observations support the hypothesis that “signal strength” is involved in MZ B cell fate decisions. The size of the MZ pool, however, does not necessarily correlate with the functionality of the MZ B cells within this splenic region. For example, while the lymphocyte adaptor protein (Lnk) transgenic mouse, the Friend leukemia virus integration 1 (Fli-1)^ΔCTA^ mouse and IL7-deficient mice have elevated MZ cell populations accompanied by an enhanced response to TNP-Ficoll [48]–[50], 3BP2-deficient mice have an increased MZ splenic compartment but manifest a poor TI-2 response [51]. *HIPK1^−/−^* mice have normal BM B cell development but reduced T1 and FO populations and an expanded MZ B cell population; however, *HIPK1^−/−^* mice are hyporesponsive to anti-IgM stimulation and immunization with TI-2 antigen. Thus, the *HIPK1^−/−^* phenotype also supports the role of weak BCR signaling in commitment to the MZ B cell fate.

B1 B cells function in a similar manner as MZ B cells in that they are a source of natural antibodies, are rapidly activated, and are involved in the TI-2 humoral response [52]. Though the “signal strength” hypothesis would predict a decrease in the *HIPK1^−/−^* B1 B cell population, this is not the case. The B cell phenotype observed in *HIPK1^−/−^* mice is similar to the phenotype observed in the *Fli-1^ΔCTA^* mice, which express a truncated Fli-1 lacking the C-terminal transcriptional activation domain [50]. Both strains have a diminished FO B population in the presence of an increased MZ B cell population, with an unperturbed peritoneal B1 population. The preservation of the B1 population in both of these genetic models may reflect adaptation to reduced B cell numbers consistent with the “production bottleneck” hypothesis.

Perhaps the most striking aspect of the *HIPK1^−/−^* immune phenotype is the impaired response to the TI-2 antigen TNP-Ficoll. *HIPK1^−/−^* mice harbour a greater number of MZ B cells compared to wild-type controls, which would suggest a normal or heightened response to TI-2 antigens. However, *HIPK1^−/−^* MZ B cells, though abundant in number, were hyporesponsive to TNP-Ficoll. This may reflect the impaired BCR-responsiveness that is driving commitment to the MZ subset. The function of the marginal zone macrophages may be impaired in the absence of *HIPK1*, contributing to the hyporesponsiveness to TNP-Ficoll, and requires further study. Previous reports have demonstrated that in the absence of HIPK family members, proliferative capacity is impaired [23], [39], [53]. Iacovelli *et al*. reported that HIPK2 is undetectable in resting human peripheral blood lymphocytes, but is reactivated in proliferating cells. Depletion of HIPK2 in immortalized human fibroblasts and mammary cells by siRNA resulted in greatly impaired proliferation and induction of p21*^waf-1/Cip-1^*, with similar findings in primary murine BM cells [53]. Thus, HIPK2 expression is positively associated with cell proliferation. The data presented in this report further supports a positive role for the HIPK family in non-damaging, cell proliferation in lymphocytes.

Since HIPKs regulate a number of transcription factors, the B cell phenotype observed in the *HIPK1^−/−^* mice may be due to a failure to optimally execute the B cell transcriptional program. Such transcriptional dysregulation may result in a switch towards MZ B cell commitment or loss of expression of genes required for optimal BCR signaling. The HIPKs are transcriptional co-regulators that can activate or repress the expression of specific target genes via phosphorylation of transcription factors and co-factors. The HIPKs have been shown to interact with several transcription factors and co-factors including p53, PAX6, RUNX1, Brn3, NK3, Tcf3 and c-Myb, Groucho, CtBP1, p300, CBP, c-Ski, Smad 1–4, MeCP2 and HMGA1 [6], [19], [20], [23], [24], [26], [29], [54]–[57]. In the case of c-Myb, HIPK1 interacts with both the N- and C-terminal ends of c-Myb, making it plausible for HIPK1 to influence c-Myb transcriptional activity in multiple ways. Tissue-specific inactivation of c-Myb in mouse B cells has demonstrated that c-Myb is involved in multiple stages of B lymphopoiesis, including the maintenance of normal splenic B cell homeostasis and proliferation [58], [59].

The HIPK family, originally identified as transcriptional co-repressors [15], are now known to regulate myriad cellular functions including embryonic development, cell survival in adult tissues, proliferation and differentiation. This study is the first to report a role for HIPK in splenic B cell homeostasis and activation.

## Supporting Information

Figure S1 ***HIPK1^−/−^*** **mice exhibit normal splenic T1 B cell viability.** To detect active caspase 3 in T1 B cells, total splenocytes were isolated from *HIPK1^+/+^* and *HIPK1^−/−^* mice and immediately incubated with FITC-DEVD-FMK (EMDBiosciences, Mississauga, ON) according to the manufacturer's instructions. The splenocytes were then stained with B220, CD23, and CD21, and 95000 total events were acquired. Cells that were B220+, CD23−, and CD21− were considered to be T1 B cells, and were measured for active caspase 3. The FACS plots are representative plots. The average percentage from three independent experiments, each done in triplicate, are shown in the bar graph.
(TIF)
