# Supplementary figures and images for: Homeodomain-Interacting Protein Kinase (HIPK)-1 Is Required for Splenic B Cell Homeostasis and Optimal T-Independent Type 2 Humoral Response
Source: PLoS One. 2012 Apr 24;7(4):e35533. doi: 10.1371/journal.pone.0035533 (PMC3335840; doi:10.1371/journal.pone.0035533)

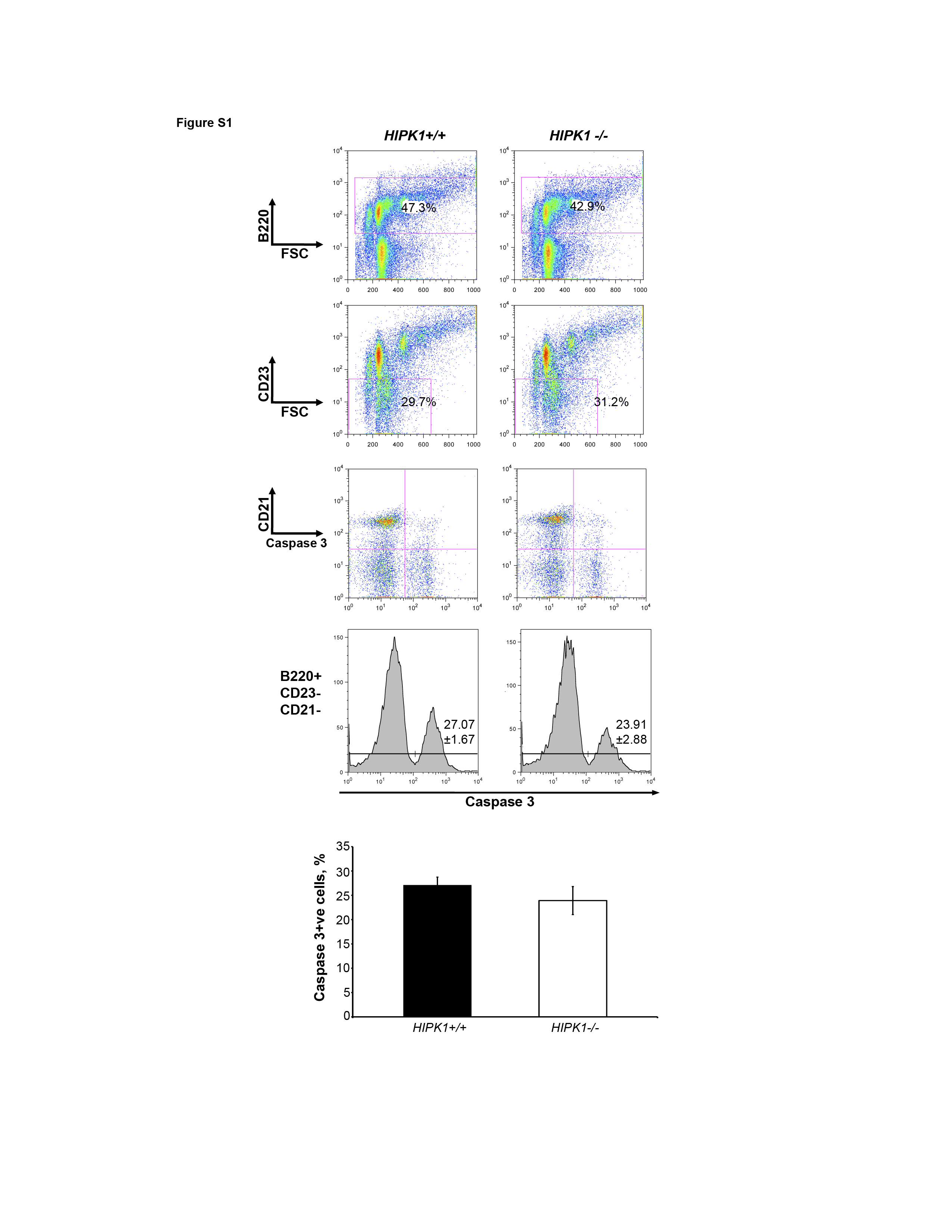

Supplement: Figure S1 — HIPK1−/− mice exhibit normal splenic T1 B cell viability. To detect active caspase 3 in T1 B cells, total splenocytes were isolated from HIPK1+/+ and HIPK1−/− mice and immediately incubated with FITC-DEVD-FMK (EMDBiosciences, Mississauga, ON) according to the manufacturer's instructions. The splenocytes were then stained with B220, CD23, and CD21, and 95000 total events were acquired. Cells that were B220+, CD23−, and CD21− were considered to be T1 B cells, and were measured for active caspase 3. The FACS plots are representative plots. The average percentage from three independent experiments, each done in triplicate, are shown in the bar graph. (TIF) [file pone.0035533.s001.tif]
